# Monomorphic Epitheliotropic Intestinal T-Cell Lymphoma in Asia Frequently Shows *SETD2* Alterations

**DOI:** 10.3390/cancers12123539

**Published:** 2020-11-27

**Authors:** Sakura Tomita, Yara Yukie Kikuti, Joaquim Carreras, Rika Sakai, Katsuyoshi Takata, Tadashi Yoshino, Silvia Bea, Elias Campo, Edoardo Missiaglia, Justine Bouilly, Audrey Letourneau, Laurence de Leval, Naoya Nakamura

**Affiliations:** 1 Department of Pathology, School of Medicine, Tokai University, 143 Shimokasuya, Kanagawa, Isehara 259-1193, Japan; ki285273@tsc.u-tokai.ac.jp (Y.Y.K.); joaquim.carreras@tokai-u.jp (J.C.); naoya@is.icc.u-tokai.ac.jp (N.N.); 2 Department of Oncology, Kanagawa Cancer Center, Kanagawa, Yokohama 241-8515, Japan; sakair@kcch.jp; 3 Department of Pathology, Graduate School of Medicine, Dentistry and Pharmaceutical Sciences, Okayama University, Okayama 700-8558, Japan; katsuyoshi.t@h5.dion.ne.jp (K.T.); yoshino@md.okayama-u.ac.jp (T.Y.); 4 Department of Pathology, Hospital Clinic Barcelona, Institut d’Investigacions Biomediques August Pi i Sunyer (IDIBAPS), Centro de Investigacion Biomedica en Red de Cancer (CIBERONC), University of Barcelona, Rossello 149-153, 08036 Barcelona, Spain; sbea@clinic.cat (S.B.); ecampo@clinic.cat (E.C.); 5 Institute of Pathology, Lausanne University Hospital, Lausanne University, Vaud, 1015 Lausanne, Switzerland; Edoardo.missiaglia@chuv.ch (E.M.); Justine.bouilly@chuv.ch (J.B.); Audrey.Letourneau@chuv.ch (A.L.); Laurence.DeLeval@chuv.ch (L.d.L.)

**Keywords:** genome profile, JAK/STAT pathway, MEITL, NGS, *SETD2*, mutational landscape, copy-number changes

## Abstract

**Simple Summary:**

Monomorphic epitheliotropic intestinal T-cell lymphoma (MEITL) is a rare primary T-cell lymphoma of the digestive tract that is characterized by an aggressive clinical course. The aim of this study was to analyze the clinicopathological characteristics and genomic profile of Asian MEITL. In this study, nine cases of Japanese MEITL were analyzed by targeted Next Generation Sequencing and immunohistochemistry and were integrated with previously reported whole-genome copy number microarray-based assay data. All cases showed alterations of the tumor suppressor gene *SETD2* and mutations in one or more genes of the JAK/STAT pathway. Therefore, we concluded that the combination of epigenetic deregulation and cell signaling activation may represent a major oncogenic event in the pathogenesis of Asian MEITL, similar to Western MEITL.

**Abstract:**

Monomorphic epitheliotropic intestinal T-cell lymphoma (MEITL) is a rare primary T-cell lymphoma of the digestive tract derived from intraepithelial lymphocytes and characterized by an aggressive clinical course. In this study, nine cases of Japanese MEITL were analyzed by targeted Next Generation Sequencing (NGS) and immunohistochemistry and were integrated with previously reported whole-genome copy number microarray-based assay data. The highlight of our findings is that all cases showed alterations of the tumor suppressor gene *SETD2* by mutations and/or loss of the corresponding 3p21 locus. We also demonstrated that all cases showed mutations in one or more genes of JAK/STAT pathway. Therefore, the combination of epigenetic deregulation and cell signaling activation represent major oncogenic events in the pathogenesis of MEITL in Asian MEITL, similar to Western MEITL.

## 1. Introduction

Monomorphic epitheliotropic intestinal T-cell lymphoma (MEITL), as described in the WHO classification of tumours of haematopoietic and lymphoid tissues, revised 4th edition [1], is a rare primary T-cell lymphoma of the digestive tract that was formerly known as Type 2 enteropathy-associated T-cell lymphoma (EATL). MEITL was first reported by Chott et al. as a CD56^+^ intestinal T-cell lymphoma with histological features of monomorphic proliferation of small to medium sized T-cells [2]. MEITL is derived from intraepithelial lymphocytes [1] and it mainly involves the jejunum and ileum; and rarely the duodenum, stomach, and colon [3], and characterized by multiple ulcerative lesions in the intestinal mucosa. Most patients are diagnosed with resected or biopsied materials by urgent surgical management due to complications such as perforation, bleeding, or obstruction [4,5]. The patients with MEITL have an aggressive clinical course and very poor prognosis. To date, no standard treatment protocols have been established.

In the past WHO classification, there were two subtypes of EATL [6]. Type 1 EATL is more prevalent in Western countries such as in Northern Europe and United States and it is strongly associated with Celiac disease (CD) [3]. Type 1 EATL is characterized by a diffuse proliferation of large-sized or anaplastic lymphoma cells with abundant intraepithelial lymphocytes, and the lymphoma cells have a CD3(+), CD8(−), CD56(−), and cytotoxic markers (+) phenotype. Type 2 EATL, on the other hand, is not associated with CD and it is distributed all over the world and represents the most prevalent form of primary intestinal T-cell lymphoma in Asia [7,8]. The histology of MEITL shows a uniform proliferation of medium to large-sized lymphoma cells with epitheliotropism. The phenotype of MEITL is of CD3(+), CD8(+), CD56(+), and megakaryocyte-associated tyrosine kinase (MATK)(+) [2,9,10,11]. Thus, in addition to the absence of prior enteropathy and the predominance in Asia, there are differences in morphology and immunophenotype with Type 2 EATL [7,11,12]. Based on these observations, MEITL was proposed as a new disease name instead of Type 2 EATL [1].

The genomic profile of MEITL have been previously reported [12,13,14,15,16,17]. Although EATL and MEITL have different morphology and phenotype, both share some common recurrent chromosomal imbalances of the copy number gains of 9q34 [13,14] and chromosome 7 and the losses of 8p22-23, 11q14 and 16q12 [13,14]. The 9q34 gain was found irrespective of TCR expression of the lymphoma cells [15,18]. MEITL is more characterized by frequent gains of the *MYC* oncogene locus (8q24) and less frequent gains of 1q and 5q loci [13,14].

Moreover, recent studies have described the molecular landscape of MEITL showing the pivotal role of *SETD2*, a tumor suppressor gene, as well as the JAK-STAT pathway [12,16]. In this pathway, *STAT5B* was altered in up to 63% of the patients. The other commonly mutated genes were *JAK3* and *SH2B3*. Those mutations provided evidence that alterations of the epigenetic deregulation and cytokine signaling pathways may have an important role in the lymphomagenesis of MEITL [12,16,17,19].

In the present study, we analyzed the alterations of *SETD2* as well as other genes associated with the JAK-STAT pathway and demonstrated that *SETD2* alterations and mutations of JAK-STAT pathway were also frequently found in Asian MEITL. In addition, we showed that *JAK3* was the most frequently mutated gene among the JAK/STAT pathway in Asian MEITL.

## 2. Results

### 2.1. Summary of Clinicopathological and Immunohistochemical Profiles

None of the MEITL cases had a history of CD. In summary, the median age of patients was 64 and the male/female ratio was 0.4. The tumor location was the small intestine in 9 cases, large intestine in 1 case, and small and large intestine in 1 case. The tumor cell size was medium to large in 8 cases, and small to medium in 3 cases. Most cases showed a CD3(+), CD5(−), CD8(+), CD56(+), and TIA-1(+) phenotype. There were 2 cases with αβ-T cell origin and 9 cases with γδ-T cell origin (Table 1).

### 2.2. Targeted NGS

**Figure 1:**
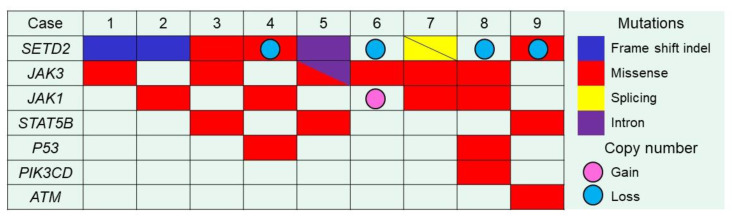
Mutations of *SETD2*, genes of JAK/STAT pathway and others. The mutational landscape of Japanese MEITL was analyzed targeting 26 genes and our previous array CGH data was included in the analysis as well. Biallelic or double mutations are represented as triangles and single mutations as full colored squares. Colored circles indicate copy-number changes.

Targeted NGS was succeeded in nine samples (cases No.1–9) among 11 samples tested with a mean coverage of 958X (range: 403X–1883X). *SETD2* was the most recurrently mutated gene, in 78% (7/9) of MEITL tumors with 8 distinctive mutations (Figure 1, Table 2, Table S1). There were frameshift indel (1 case), inframe indel (1 case), and splicing mutations (double mutations in 1 case) expected to confer critical changes in the protein structure or function. Missense mutations (i.e., change in one amino acid in a protein, arising from a point mutation in a single nucleotide) were seen in 3 cases, and in 1 of them it was predicted to be deleterious with a damaging effect by the SIFT algorithm. We found that all nine MEITL cases examined by NGS showed mutations in 1 or more genes of the JAK/STAT pathway: *JAK1*, *JAK3*, *STAT5B* mutations were identified in 4 cases (44%), 6 cases (67%), and 3 cases (33%), respectively. We found several hotspot mutations in JAK/STAT pathway. *JAK3* was the most frequently mutated gene (67%) in the pathway and included 2 hotspot mutations (Table 2). Among *JAK3* mutated cases, p.Ala573Val and p.Val674Ala were seen in 3/6 cases (50%) and 2/6 cases (33%), respectively. Both mutations are in the pseudokinase domain. Among *STAT5B* mutated cases, p.Ala642His (SH2 domain) was seen in 2/3 cases (67%). Alterations of other genes were also found, *TP53* in 2 cases (22%), *PIK3CD* in 1 case (11%), and *ATM* in 1 case (11%). There were no mutations in any of the other genes in the panel.

### 2.3. Immunohistochemistry of H3K36me3

**Figure 2:**
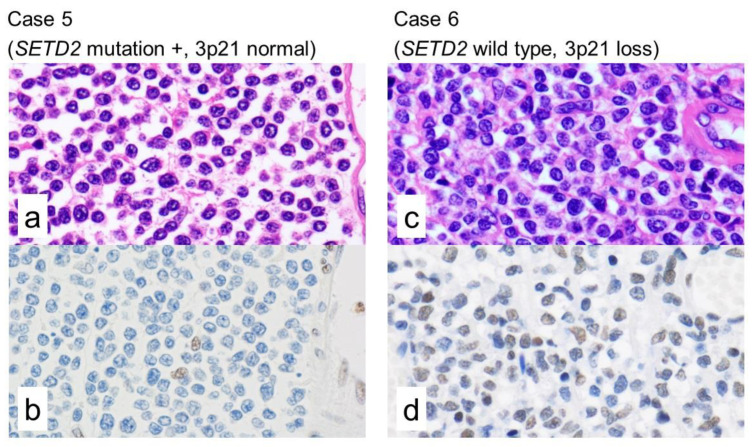
Morphology and immunohistochemical staining for H3K36me3. H3K36me3 is an epigenetic modification to the DNA packaging protein Histone H3. It is a mark that indicates the tri-methylation at the 36th lysine residue that, among other functions, maintain the gene expression stability. The trimethylation is catalyzed by SETD2, that acts as a tumor suppressor gene (TSG). Among the nine examined cases by IHC, all the seven *SETD2*-mutated cases were negative for H3K36me3. Only cases 6 and 8 had no *SETD2* mutation. Case 5 (*SETD2* mutation and normal copy number of 3p21) showed negativity for H3K36me3. Case 6 (no mutations in *SETD2* and loss of 3p21) showed positivity for H3K36me3. ((**a**,**c**): hematoxylin and eosin, (**b**,**d**): H3K36me3).

*SETD2* encodes a methyltransferase that in humans is non-redundantly responsible for specifically trimethylating lysine 36 of histone H3 (marked as H3K36me3) using the dimethylated lysine 36 (H3K36me2) as a substrate [20]. Therefore, the H3K36me3 is an epigenetic modification to the DNA packaging protein Histone H3. This histone methylation is responsible for maintaining gene expression stability. To determine the functional consequences of *SETD2* alterations, we performed IHC for H3K36me3. The seven cases with *SETD2*-mutation were negative for H3K36me3. In the other two cases, 3p21.31 loss by microarray and no *SETD2* mutation, H3K36me3 was positive in case 6 and negative in case 8 (Figure 2).

### 2.4. Integrated Analysis of Targeted NGS, a Whole-Genome Copy Number Microarray-Based Assay and Immunohistochemistry

We performed integrated analysis of targeted NGS, a whole-genome copy number microarray-based assay (Oncoscan analysis) and immunohistochemistry for seven cases (Table 2, Figure 1). The Oncoscan analysis data was retrieved from our previous publication (Figure S1) [15]. Our cases showed deletion of the *SETD2* locus (3p21.31) in 4/7 cases (57%). Among those 4 cases, 1 had a pathogenic missense mutation in *SETD2*, 1 had a missense mutation with unclear pathogenicity, and 2 had no detectable *SETD2* mutation. Two of the remaining 3 cases had a pathogenic mutation (frameshift mutation and mutation in intron, not in splicing site), 1 had a missense mutation with unclear pathogenicity. Regarding the JAK/STAT pathway, the loci of genes were altered in some cases, the gains of 1p31.3 (*JAK1*), 19p13.11 (*JAK3*), and 17q21.2(*STAT5B*) were seen in 1/8 (13%), 0/8 (0%), 1/8 (13%), respectively. No case showed copy-number loss of these loci.

## 3. Discussion

In this study, the mutational landscape by NGS of nine cases of Japanese MEITL was analyzed. The highlight of integrated analysis of NGS, a whole-genome copy number microarray-based assay and immunohistochemistry is the finding of alterations of *SETD2* gene by mutations and/or loss of the corresponding 3p21 locus in all cases.

SETD2 is a histone methyltransferase that specifically regulates tri-methylation at the 36th lysine residue of the histone H3 protein (marked as H3K36me3) [21]. Methylation of this residue is associated with active chromatin. Specifically, SETD2-mediated H3K36me3 is associated with DNA double strand breaks, active transcription, homologous recombination repair, and genome stability [22,23,24]. Pathogenic *SETD2* mutations have been detected in several cancers, including renal cell carcinomas, breast carcinomas, lung cancers, central nervous system tumors, leukemia [25,26,27,28], and lymphoma in particular MEITL and hepatosplenic T-cell lymphoma [29]. *SETD2* inactivation has been shown to be a critical event in facilitating both disease initiation and progression through decreasing H3K36me3 [30]. So far, highly recurrent *SETD2* alterations in MEITL (i.e., Type 2 EATL) have been reported by two groups: this was first described by Roberti et al., who reported alterations in *SETD2* gene in 93% of western European cases (14/15), mainly as loss of function mutations and/or loss of the corresponding 3p21.31 locus [12], and subsequently by Moffitt et al., who found *SETD2* mutations in 70% of cases from North America (16/23) [17].

Our study confirms a highly prevalent *SETD2* alteration in all Japanese MEITL. The mutations in our cases were missense (3 cases), frameshift indel (1 cases), Inframe indel (1 case), splicing mutation (1 case), and mutation in intron (1 case). The frameshift indels predict to impair protein function. Other mutations can be evaluated using SIFT as this software predicts whether an amino acid substitution affects protein function based on sequence homology and the physical properties of amino acids. According to SIFT, 5/7 (71%) *SETD2*-mutated cases were thought to be pathogenic, and in the remaining 2/7 (29%), the pathogenesis was uncertain. Loss of heterozygosity (LOH) is a cross chromosomal event that results in loss of the entire gene and the surrounding chromosomal region. The LOH is a common occurrence in cancer, where it indicates the absence of a functional TSG in the lost region. Then, the remaining copy of the TSG can be inactivated by a point mutation. In accordance with the notion that biallelic hits on the same gene are generally required for inactivating the function of a TSG, we found two-hits (biallelic) in *SETD2* in 2 cases (case 4 and 9) with 3p21.31 loss and mutation, but in 7 cases we could not confirm biallelic *SETD2* alteration. By IHC, all 7 *SETD2* mutated cases, not only two-hit alterations but also single-hit *SETD2* mutations, showed complete loss of H3K36me3. It is reported that monoallelic *SETD2* mutations impair H3K36me3 by other groups, and the notion of a dominant phenotype is considered [12,31,32]. H3K36me3 was expressed in case 6 (*SETD2* wild type and loss of 3p21). On the other hand, case 8 was negative for H3K36me3 by IHC; nevertheless, it had the same *SETD2* alteration. In clear cell renal cell carcinoma, it is demonstrated that total H3K36me3 levels are not significantly impacted by monoallelic loss of *SETD2* [33]. Therefore, our case 6 might have monoallelic loss of *SETD2*, not biallelic. In the array CGH data, the degree of 3p21 deletion was not so different between case 6 and case 8, and it is difficult to think that case 8 had biallelic loss of 3p21. In general, abnormal methylations are also associated with loss of the function of TSG, and the absence of H3K36me3 in case 8 might be caused by epigenetic abnormality. Recently, H3K36me3-deficient cancers can be selectively targeted by inhibition of WEE1, a cell cycle controlling kinase [34]. WEE1 inhibitor may be the new therapeutic option for MEITL, which has no established method of treatment.

Moreover, we found mutations that can potentially induce deregulation and activation of the JAK-STAT pathway in Japanese MEITL. This pathway is involved in the function of many cytokines and it plays a role in the oncogenic mechanism of several T and NK leukemia and lymphomas [19,35,36,37,38]. We found that all Japanese MEITL cases had mutations in one or more genes of the JAK/STAT pathway, which is also known to regulate the intraepithelial lymphocyte function [39,40,41]. Recurrent alterations of the JAK/STAT pathway in MEITL have been reported by several groups, including an Asian group [12,16,17]. We confirmed that mutations affecting the JAK/STAT pathway are not mutually exclusive. This observation is in accordance with findings reported by Roberti et al. [12]. We found mutations in *JAK3* (6/9 cases, 67%), which was the most frequently mutated gene in the pathway. *JAK3* mutations are reported to be seen in 35–46% of MEITL by other groups, and they showed that the *STAT5B* mutations are more frequent (60–63%) [12,16,17]. The frequency of *JAK3* mutations were especially high in our cases, compared with other groups. The hotspots of *JAK3* mutations (p.Ala573Val and p.Val674Ala) were in concordance with the findings reported by Nairismägi et al. [16]. *JAK3* p.Ala573Val mutation is also reported in Extranodal NK/T-cell lymphoma, nasal type [42,43,44], and mycosis fungoides [45]. In this study, *STAT5B* mutations were seen in 3/9 cases, 33%. p.Ala642His was identified as a hotspot, but the mutation has not been reported in any tumors until now. Finally, we found high frequency of mutations in the JAK/STAT pathway as in the European MEITL. Therefore, the mutations of the JAK/STAT pathway seem to be a universal feature in MEITL lymphomagenesis. However, there was a different point that *JAK3* was the most frequently mutated gene in the pathway.

## 4. Materials and Methods

### 4.1. Cases and Materials

We used a total of 11 formalin-fixed paraffin-embedded (FFPE) samples of Japanese MEITLs that were previously reported [15]. FFPE samples were obtained from surgical resections in 10 patients and a tumor biopsy in 1 patient.

### 4.2. Targeted NGS

The suitability of the material for molecular analysis was assessed and the areas with highest tumor cell content were marked on the hematoxylin-eosin (HE) control slides by a pathologist (ST). After staining with toluidine blue, the slides were manually microdissected by scraping the areas of interest of the tissue under the microscope to select the areas of highest tumor cell content. This was followed by a genomic DNA extraction using the QIAamp DNA FFPE Tissue Kit (#56404, Qiagen AG) on an automatic sample processing equipment (QIAcube, Qiagen AG, Garstligweg, Switzerland).

High-throughput sequencing analysis [i.e., Next-generation sequencing (NGS)] was performed on a MiSeq^®^ System platform (Illumina, Inc., San Diego, CA, USA) using the xGen^®^ Lockdown^®^ IDT Custom T-cell Lymphoma Panel (Integrated DNA Technologies, Inc., Coralville, IA, USA) at the Institute of Pathology of Lausanne University Hospital, as previously described [46]. The panel covered the complete coding sequences of 26 genes of interest in peripheral T-cell lymphomas: *ARID1A*, *ATM*, *BCOR*, *CARD11*, *CCR4*, *CD28*, *CTNNB1*, *DDX3X*, *DNMT3A*, *FYN*, *IDH2*, *IRF4*, *JAK1*, *JAK3*, *KMT2D*, *PIK3CD*, *PLCG1*, *PRKCB*, *RHOA*, *SETD2*, *STAT3*, *STAT5B*, *TET2*, *TNFRSF1B*, *TP53,* and *VAV1*. Burrows-Wheeler Aligner software (repository: https://sourceforge.net/projects/bio-bwa/) was employed to align sequence reads to the hg19 human genome assembly, followed by re-alignment around indels and base quality re-calibration, in compliance with GATK best practices (https://software.broadinstitute.org/gatk/best-practices/). Variants (point mutations and small insertions and deletions (indels)) were detected combining calls from both VarScan (v.2.4.2) and MuTect2 (GATK v.3.7) variant callers packages using default settings. Filter were applied to select variants based on local coverage (> 50), number of altered reads (> 5), allele frequency (> 1%), and strand bias. Variants were annotated using SnpEff (v.4.3) and following the recommendations of the Human Genome Variation Society (HGVS). The prediction of the functional significance of the identified variants was assessed using the Sorting Intolerant from Tolerant (SIFT), Clin Var, OncoKB. Leiden Open Variation Database (LOVD), Clinical Knowledgebase (CKB) prediction algorithm software and available literature.

The whole-genome copy-number profile was obtained from our previous publications [15]. In summary, DNA had been extracted using the Qiagen DNA extraction kit and the profile was obtained using an array CGH kit of Agilent SurePrint G3 Human CGH (Agilent K.K., Tokyo, Japan).

### 4.3. Immunohistochemistry

Immunohistochemistry was performed using an automated Stainer (Bond-max autostainer, Leica Microsystems K.K., Tokyo, Japan) with a rabbit polyclonal antibody for detection of methylated Histone H3 (tri methyl K36, H3K36me3, #ab9050, Abcam, Cambridge, UK) at 1:2000 dilution and BOND IHC Polymer Detection Kit (#DS9800, Leica Microsystems K.K.). H3K36me3 marker was evaluated by three pathologists (ST, JC, NN) who were blinded to the *SETD2* genotype. The samples were judged positive if the H3K36me3 positivity was equivalent to that of the internal positive controls including vascular endothelial cells, macrophages, and reactive lymphocytes; and they were assessed as negative if the tumor cells were not stained at all.

### 4.4. Ethics

This study was approved by the institutional review board of the participating institutions where required (14R-079), was conducted in accordance with the Helsinki Declaration of 1975 as revised in 2013, and in accordance with the ethical standards on human experimentation. There is compliance with The Biospecimen Reporting for Improved Study Quality (BRISQ), Minimum Information about a Microarray Experiment (MIAME), and Reporting Recommendations for Tumor Marker Prognostic Studies (REMARK) guidelines.

## 5. Conclusions

In conclusion, our study disclosed the genetic profile of Asian MEITL characterized by *SETD2* alterations (either single or double hit/biallelic) and mutations affecting the JAK/STAT pathway in concordance with Western cases. All these gene abnormalities might have a pathogenic role of MEITL.

## Figures and Tables

**Table 1:** Clinical features of the patients and T-cell receptor (TCR) status.

Case	Age	Sex	Celiac Disease	Location	Treatment	Prognosis (Survival Time)	TCR
1	66	Female	No	Small and Large intestine	Operation	Unknown	γδ
2	64	Male	No	Small intestine	Operation, DeVIC	Dead (Unknown)	γδ
3	58	Male	No	Small intestine	Operation	(Unknown)	γδ
4	74	Female	No	Small intestine	Operation, CHOP	Unknown	γδ
5	49	Female	No	Small intestine	Operation, CHO, GEM	Dead (6 months)	γδ
6	70	Female	No	Small intestine	Operation	Alive (4 months)	γδ
7	62	Female	No	Small intestine	Operation, CHOP	Dead (7 months)	γδ
8	61	Female	No	Large intestine	Operation, DeVIC	Alive (50 months)	γδ
9	81	Female	No	Small intestine	Operation, THP-COP, CDE-11	Dead (8 months)	αβ
10	64	Female	No	Small intestine	Operation, EPOCH	Unknown	γδ
11	75	Male	No	Small intestine	Operation, CHO	Dead (3 months)	αβ

The data of TCR are from reference [18]. Abbreviations: DeVIC (carboplatin, ifosfamide, etoposide, and dexamethasone); CHOP (cyclophosphamide, doxorubicin, vincristine, and prednisolone); CHO (cyclophosphamide, doxorubicin, and vincristine); GEM (gemcitabine); THP-COP (cyclophosphamide, pirarubicin, vincristine, and prednisolone); CDE-11 (irinotecan, carboplatin, etoposide, and dexamethasone); EPOCH (etoposide, vincristine, doxorubicin, cyclophosphamide, and prednisolone).

**Table 2:** Summary of DNA sequencing and IHC of H3K36me3.

Case	Gene	Mutation Type	CNV	Exon	AA Changes	SIFT Prediction	H3K36me3 IHC	COSMIC ID
1	*SETD2*	Inflame indel	NA	exon 20	p.Phe2481-Gln2484del	Probably damaging	Negative	None
	*JAK3*	Missense	NA	exon 13	p.Ala573Val	Damaging	-	COSM34215(17)
2	*SETD2*	Frameshift indel	Normal	exon 20	p.Tyr2489Trpfs*6	Probably damaging	Negative	None
	*JAK1*	Missense	Normal	exon 22	p.Lys1026Glu	Probably damaging	-	None
3	*SETD2*	Missense	Normal	exon 7	p.Cys1631Phe	Incertain	Negative	None
	*JAK3*	Missense	Normal	exon 13	p.Ala573Val	Damaging	-	COSM34215(17)
	*STAT5B*	Missense	Normal	exon 18	p.Val712Glu	Damaging	-	COSM5414165(1)
4	*SETD2*	Missense	Loss	exon 20	p.Leu2486Arg	Probably damaging	Negative	None
	*JAK1*	Missense	Normal	exon 15	p.Ser703Ile	Damaging	-	COSM305942(10)
	*TP53*	Missense	Normal	exon 7	p.Gly244Asp	Damaging	-	COSM10735(43)
5	*SETD2*	Substitution (intron)	Normal	exon 5	-	Probably damaging	Negative	None
	*JAK3*	Splicing	Normal	exon 20	-	Incertain	-	None
		Missense		exon 13	p.Ala573Val	Damaging	-	None
	*STAT5B*	Missense	Normal	exon 16	p.Asn642His	Damaging	-	COSM1716590(29)
6	*SETD2*	WT	Loss	-	-	-	Positive	None
	*JAK3*	Missense	Normal	exon 11	p.Met511Ile	Probably damaging	-	COSM51374(29)
7	*SETD2*	Splicing	NA	exon 1	-	Probably damaging	Negative	None
		Splicing		exon 6	-	Probably damaging		None
	*JAK1*	Missense	NA	exon 17	p.Leu783Phe	Probably damaging	-	COSM41758(5)
	*JAK3*	Missense	NA	exon 15	p.Val674Ala	Probably damaging	-	COSM327317(7)
8	*SETD2*	WT	Loss	-	-	-	Negative	None
	*JAK1*	Missense	Normal	exon 15	p.Ser703Cys	Incertain	-	None
	*JAK3*	Missense	Normal	exon 15	p.Val674Ala	Probably damaging	-	COSM327317(7)
	*PIK3CD*	Missense	Normal	exon 8	p.Met339Lys	Incertain	-	None
	*TP53*	Missense	Normal	exon 10	p.Arg337His	Probably damaging	-	None
9	*SETD2*	Missense	Loss	exon 11	p.Ser1769Tyr	Incertain	Negative	None
	*STAT5B*	Missense	Normal	exon 16	p.Asn642His	Damaging	-	COSM1716590(29)
	*ATM*	Missense	Normal	exon 19	p.Pro960His	Incertain	-	None

The data of CNV are from reference [15]. Abbreviations: CNV, copy number variation; COSMIC, Catalogue of Somatic Mutation in Cancer; NA, not analysed.

## Supplementary Materials

The supplementary materials are available online at https://www.mdpi.com/2072-6694/12/12/3539/s1, Table S1: Summary of DNA sequencing and IHC of H3K36me3, listed by gene symbol, Figure S1: Summary view of the copy-number alterations across the genome (from [15]).
