# Supplementary material for: Monomorphic Epitheliotropic Intestinal T-Cell Lymphoma in Asia Frequently Shows SETD2 Alterations
Source: Cancers (Basel). 2020 Nov 27;12(12):3539. doi: 10.3390/cancers12123539 (PMC7759862; doi:10.3390/cancers12123539)
Supplement: Supplementary file 1 [file cancers-12-03539-s001.pdf]

Supplementary Materials:

Monomorphic Epitheliotropic Intestinal T-Cell Lymphoma in Asia Frequently Shows *SETD2* Alterations

Sakura Tomita [1,*], Yara Yukie Kikuti [1], Joaquim Carreras [1], Rika Sakai [2], Katsuyoshi Takata [3], Tadashi Yoshino [3], Silvia Bea [4], Elias Campo [4], Edoardo Missiaglia [5], Justine Bouilly [5], Audrey Letourneau [5], Laurence de Leval [5] and Naoya Nakamura [1]

Table S1. Summary of DNA sequencing and IHC of H3K36me3, listed by gene symbol.

Case	*SETD2*			*JAK1*		*JAK3*		*STAT5B*		Others	
	Mutations	CNV	IHC	Mutations	CNV	Mutations	CNV	Mutations	CNV	Mutations	CNV
1	Inframe indel exon 20 p. Phe2481_Gln2484del (Probably damaging)	NA	Negative	WT	NA	Missense exon 13 p. Ala573Val (Damaging)	NA	WT	NA		
2	Frameshift indel exon 20 p. Tyr2489Trpfs*6 (Probably damaging)	Normal	Negative	Missense exon 22 p. Lys1026Glu (Probably damaging)	Normal	WT	Normal	WT	Gain		
3	Missense exon 7 p. Cys1631Phe (Incertain)	Normal	Negative	WT	Normal	Missense exon 13 p. Ala573Val (Damaging)	Normal	Missense exon 18 p. Val712Glu (Damaging)	Normal		
4	Missense exon 20 p. Leu2486Arg (Probably damaging)	Loss	Negative	Missense exon 15 p. Ser703Ile (Damaging)	Normal	WT	Normal	WT	Normal	*TP53* Missense exon 7 p. Gly244Asp (Damaging)	Normal
5	Substitution (intron) exon 5 c. 4715+1G>T (Probably damaging)	Normal	Negative	WT	Normal	Splicing exon 20 c. 2805+7G>C (Incertain)	Normal	Missense exon 16 p.	Normal		

						Missense<br>exon 13<br>p. Ala573Val<br>(Damaging)		Asn642His<br>(Damaging)			
6	WT	Loss	Positive	WT	Gain	Missense<br>exon 11<br>p. Met511Ile<br>(Probably damaging)	Normal	WT	Normal		
7	Splicing<br>exon 6<br>c. 4716-1G>A<br>(Probably damaging)	NA	Negative	Missense<br>exon 17<br>p. Leu783Phe<br>(Probably damaging)	NA	Missense<br>exon 15<br>p. Val674Ala<br>(Probably damaging)	Normal	WT	Normal		
	Splicing<br>exon 1<br>c. 71+1G>A<br>(Probably damaging)										
8	WT	Loss	Negative	Missense<br>exon 15<br>p. Ser703Cys<br>(Damaging)	Normal	Missense<br>exon 15<br>p. Val674Ala<br>(Probably damaging)	Normal	WT	Normal	*PIK3CD*<br>Missense<br>exon 8<br>p. Met339Lys<br>(Incertain)	Normal
										TP53<br>Missense<br>exon 10<br>p. Arg337His<br>(Probably damaging)	Normal
9	Missense<br>exon 11<br>p. Ser1769Tyr<br>(Incertain)	Loss	Negative	WT	Normal	WT	Normal	Missense<br>exon 16<br>p. Asn642His<br>(Damaging)	Normal	*ATM*<br>Missense<br>exon 19<br>p. Pro960His<br>(Incertain)	Normal

The data of CNV are from refference 15.
Abbreviations: CNV, copy nuber varidation; NA, not analysed; WT, wild type

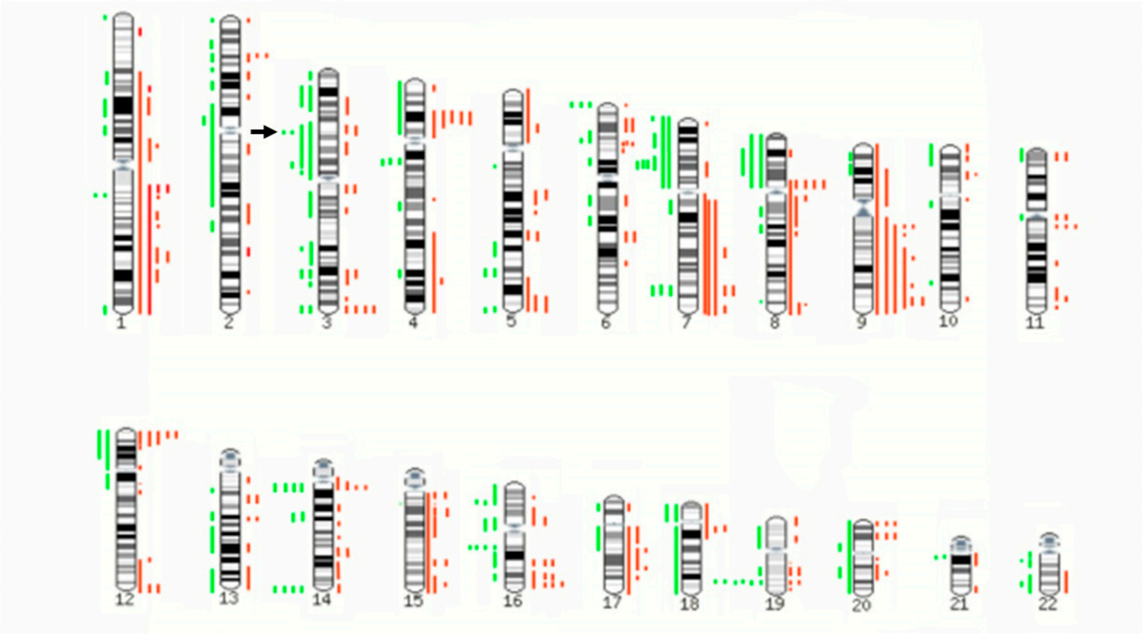

Figure S1. Summary view of the copy-number alterations across the genome (from reference 15). In the karyoview the red lines to the right of the chromosomes represent areas of gain and green lines on the left side of the chromosomes represent areas of loss. The genome profile of MEITL in Japan was characterized by multiple regions of gains and losses such as gain of 4p15.1 (5/8, 62.5%), 7q34 (5/8, 62.5%), 8p11.23 (5/8, 62.5%), 9q22.31 (5/8, 62.5%), 9q33.2 (5/8, 62.5%), 9q34.13 (6/8, 75%) and losses of 7p14.1 (6/8, 75%), 19q33.33 (6/8, 75%). Loss of *SETD2* at 3p21 in 4 cases is marked with an arrow.
